# Molecular Signaling in Tumorigenesis of Gastric Cancer

**DOI:** 10.22034/ibj.22.4.217

**Published:** 2018-07

**Authors:** Fatemeh Molaei, Mohammad Mahdi Forghanifard, Yasaman Fahim, Mohammad Reza Abbaszadegan

**Affiliations:** 1 Immunology Research Center, Mashhad University of Medical Sciences, Mashhad, Iran; 2 Department of Biology, Damghan Branch, Islamic Azad University, Damghan, Iran; 3 Medical Genetics Research Center, Mashhad University of Medical Sciences, Mashhad, Iran

**Keywords:** Beta Catenin, Epithelial-mesenchymal transition, Hedgehogs, *Helicobacter pylori*, NF-kappa B

## Abstract

Gastric cancer (GC) is regarded as the fifth most common cancer and the third cause of cancer-related deaths worldwide. Mechanism of GC pathogenesis is still unclear and relies on multiple factors, including environmental and genetic characteristics. One of the most important environmental factors of GC occurrence is infection with *Helicobacter pylori* that is classified as class one carcinogens. Dysregulation of several genes and pathways play an essential role during gastric carcinogenesis. Dysregulation of developmental pathways such as Wnt/β-catenin signaling, Hedgehog signaling, Hippo pathway, Notch signaling, nuclear factor-kB, and epidermal growth factor receptor have been found in GC. Epithelial-mesenchymal transition, as an important process during embryogenesis and tumorigenesis, is supposed to play a role in initiation, invasion, metastasis, and progression of GC. Although surgery is the main therapeutic modality of the disease, the understanding of biological processes of cell signaling pathways may help to develop new therapeutic targets for GC.

## INTRODUCTION

Gastric cancer (GC) is one of the most common and lethal cancers worldwide. More than 950,000 new cases are diagnosed annually[1]. The incidence of GC is higher in Eastern Asia, Eastern Europe, and Southern America than Northern America and Northern Africa[2]. In Iran, GC is prevalent in northern and northwestern regions, and men are twice as likely to be affected than women[3]. GC is the fourth most common cancer (after lung, prostate, and colorectal cancers) in men and the fifth most common cancer (after breast, cervical, colorectal, and lung cancers) in women globally[4]. Despite the declining rate of GC incidence and advances in diagnosis, GC causes more than 700,000 death annually, and a five-year survival rate is nearly 20%[5].

Gastric adenocarcinoma has recently been classified genetically to four molecular subtypes, including chromosomal instability, microsatellite instability, genome stable, and Epstein-Barr virus-positive[6]. There are two main histological types of GC consisting of intestinal and diffuse types. Development of the intestinal type includes the transformation of normal mucosa to the similar mucosa of the intestinal epithelium. These series of mucosal alterations are triggered by chronic inflammation (gastritis), which eventually leads to metaplasia, dysplasia, and cancer. The diffuse type appears as single-cell that changes in the mucous neck area of gastric glands[7]. Thirty to 50% of the diffuse types are caused by either point or small frameshift mutations in *CDH1* gene, which encodes E-cadherin and plays an essential role in cell adhesion[8].

Some of the main risk factors of GC are summarized in Table 1, including *Helicobacter pylori* infection and atrophic gastritis, tobacco smoking, dietary salt and food preservation, pernicious anemia, and abnormalities in E-cadherin gene[9]. The aim of this review is to summarize several important signaling pathways in GC, which helps to have a better understanding of GC biology.

**Table 1:** GC risk factors

GC risk factor	Explanations	Reference
*H. pylori* infection	Most important risk factor, long-term infection, leads to chronic atrophic gastritis and pre-cancerous alterations. The international agency for research on cancer (IARC) classified *H. Pylori* as the first class carcinogen. People with GC have a higher rate of *H. pylori* infection.	[105]
Smoking	Smoking increased the risk of GC. Studies have reported that smokers have higher hazard ratio in GC in cardia (2.86–4.10) compared with the distal region of stomach (1.52–1.94).	[106]
E-cadherin gene	Hereditary diffuse GC caused by the mutation in *CDH1* gene encodes E-cadherin.	[107]
Pernicious anemia	People with Pernicious anemia have increased the risk of GC. More studies are needed to confirm this condition.	[9]
Diet	Diet play important role in prevention and development of GC. Salt and salt-preserved foods increased the risk of GC. Intake twice or more of fruits and vegetables in a day decreased the risk of GC.	[7]
Epstein-Barr virus (EBV)	5% to 10% of GCs are associated with EBV. Its mechanism is DNA methylation (gene silencing).	[108]

### Molecular pathways of GC

There are several cell signaling pathways playing a role in gastric carcinogenesis. Here, we review different cell signaling pathways that are involved in GC tumorigenesis, highlighting either the expression pattern or contributed mutations in related genes.

#### Hedgehog (Hh) signaling pathway

**Fig. 1:**
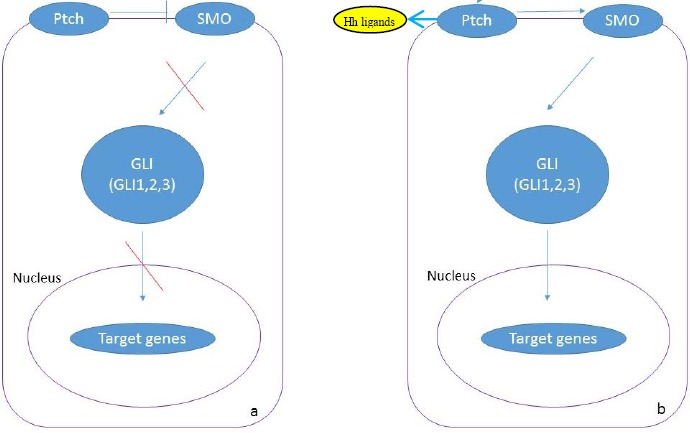
Hh pathway in Hh signaling. In the absence of ligands Ptch inhibits SMO and then inactivates the signaling pathway (a). (b) In the presence of ligands, ligands bind to the Ptch, and the activation of SMO and signaling pathway occurs (b).

The Hh signaling pathway is important in embryonic development, differentiation, proliferation, and maintenance of some adult tissues. Ligands of this pathway in mammals include Sonic, Indian, and Desert. In the absence of these ligands, the transmembrane receptor ptch inhibits another transmembrane protein (smoothened [SMO]), resulting in deactivation of Hh pathway. By binding ligands to the ptch receptor, the inhibitory effect of patch is eliminated from SMO, and SMO activates the downstream transcription factors, including GLI (GLI1, GLI2, and GLI3) proteins. Then GLI translocates to the nucleus and activates Hh-related target genes[10] (Fig. 1).

In the gastrointestinal tract, where epithelial cells are continuously replenished from progenitor cell populations, Hh signaling appears to be essential for restoration. During GC processing, chronic *H. pylori* infection causes mucosal damage. Furthermore, the overexpression of sonic Hh has been detected in progenitor cells (in gastric mucosa), which restore the damaged gastric mucosa[11]. In addition, overexpression of *GLI1* was correlated to the lymph node metastasis in esophageal squamous cell carcinoma (ESCC) patients[12]. The expression changes of this pathway in GC are summarized in Table 2.

**Table 2:** Hh pathway

Up-regulated genes	Explanations	Reference
*SHH*, *PTCH*, and *GLI1*	Up-regulation of these genes is observed during *H. pylori* infection in GC cells. CagA-positive *H. pylori* was correlated with the higher expression of *SHH*.	[11]
*PTCH1*, *SMO*, and *GLI* *Shh* and *Ihh*	Overexpression of these genes is documented in diffuse types of GC. Expression of *Shh* and *Ihh* is detected in the intestinal type of GC.	[109]
*GLI1*	The up-regulation of *Gli1* and down-regulation of *SuFu* have been reported in GC tissue. *Gli1* overexpression is correlated with aggressive phenotype.	[110]
*SHH*	*SHH* overexpression is related with age, tumor differentiation state, T staging, and N stage in GC. In another study, *SHH* expression is correlated with lymphatic metastasis and poor prognosis. Furthermore, in xenograft of human GC, the up-regulation of *SHH* significantly enhances the incidence of lung metastasis.	[111]
*SHH*, *PTCH*, and *Gli3*	The expression of these genes increases in CD44+ and CD24 + subpopulation, which is comparable with the CD44−CD24−subpopulation.	[112]

#### Wnt/β-catenin pathway

Wnt proteins are cysteine-rich glycoproteins that bind to the extracellular domain of frizzled receptor and lipoprotein receptor-related protein 5/6. Wnt signaling regulates different cellular processes, including cell fate, movement, polarity, and organogenesis. There are three types of Wnt pathways. The first is canonical or β-catenin-dependent pathway that involves in the stabilization of the proto-oncogene β-catenin. The second is planar cell polarity pathway that involves in cell ciliogenesis. The last is Wnt/Ca2+-dependent pathway that stimulates the intracellular release of calcium and activates Ca2+-dependent mediators controlling cell movement and behavior. The planar cell polarity and Wnt/Ca2+ pathways are collectively called either non-canonical or β-catenin-independent pathway[13].

In the absence of Wnt, GSK3 in APC complex (including APC, AXIN, CK1, and GSK3) phosphorylates β-catenin, which in turn leads to the degradation of β-catenin in proteasome complex. Binding of Wnt ligand to the frizzled receptor inhibits GSK3 activity through dishevelled, resulting in dephosphorylation and stabilization of the β-catenin. Therefore, β-catenin accumulates in the nucleus, and its interaction with the T-cell factor/lymphoid enhancer factor (TCF/LEF) transcription factor family stimulates the transcription of Wnt target genes[14]. Two signaling pathways, including nuclear factor **(**NF)-kB and Wnt/β-catenin are dysregulated in 70% of the GC patients[15]. Wnt pathway is a key element in cell proliferation during both normal and cancerous gut development. SALL4, as an embryonic stem cell marker, has a direct interaction with Wnt signaling. Its overexpression is correlated with lymph node metastasis in GC[16]. Furthermore, overexpression of *SALL4* and *SOX2*, members of the sex-determining region Y-related high-mobility group (HMG), are observed in ESCC, and the expression levels of these two genes are correlated with each other[17]. The overexpression of *SALL4* has also been detected in patients with colorectal cancer, and its overexpression is associated with the grade of tumor cell differentiation and tumor cell metastasis to the lymph node[18]. Some of the genetic alterations of this pathway are summarized in Table 3.

**Table 3:** Genetic alteration of Wnt pathway

Gene	Genetics alteration	Explanation	Reference
*Wnt-1*	Up-regulated		[113]
*Wnt-2*	Up-regulated	The overexpression of *WNT2* is correlated with cytoplasmic/nuclear β-catenin accumulation in both intestinal- and diffuse-type ofr GC in Chinese people. Moreover, the expression of *WNT2* positively is correlated with lymph node metastasis.	[114]
*Wnt-5*	Up-regulated	Its expression is correlated with poor prognosis.	[115]
*Fzd-3*	Up-regulated	Its overexpression is correlated with the activation of Wnt signaling in GC.	[116]
*CTNNB1*	Mutation	Mutation in the gene (*CTNNB1*) is found in diffuse and intestinal type of GC.	[117]
*TCF7L2*	Somatic frame shift mutation	Somatic frame shift mutation is detected in GC with microsatellite instability.	[118]
*APC*	Mutation	Mutation and deletion	[119]
*Sox10*	Down-regulated	*Sox10* is a transcription factor that regulates Wnt signaling.	[120]
*WNT10A*	Up-regulated	*H. Pylori* infection induces this overexpression.	[121]

*H. pylori* infection dysregulates Wnt signaling pathway. CagA, the most important virulence factor of *H. pylori*, causes the activation of the β-catenin through an independent phosphorylation manner. CagA interacts with E-cadherin, leading to β-catenin accumulation in cytoplasm and nucleus. Moreover, CagA transactivates *CDX1* and *P21* genes that are involved in the intestinal differentiation of gastric epithelial cells[19]. VacA, another *H. pylori* virulence factor, induces Wnt/β-catenin signaling through the activation of PI3K/Akt pathway, resulting in phosphorylation of GSK3β and translocation of the β-catenin to the nucleus to activate *CCND1* gene[20]. Moreover, *H. pylori* infection increases the expression levels of Oct4 and Nanog, two cancer stem cell (CSC) markers, through Wnt signaling that promotes CSC-properties in GC cells[21].

Transglutaminase (TGM) family plays an essential factor in drug resistance and progression of cancers. The expression level of *TGM1*, a member of TGM family, is elevated in GC that indicates *TGM1* participation in the development of this disease. Moreover, the reduced levels of *TGM1* in GC cells result in the suppression of Wnt signaling activities. This result suggests that the *TGM1* may function in GC by affecting Wnt signaling pathway[22].

#### Cell cycle

Dysregulation of the cell cycle components is a defining factor in gastric tumorigenesis. Activation of the cyclin-dependent kinase (CDK) results in cell cycle progression. Cyclin D1 and cyclin D2 are up-regulated in GC[23]. Furthermore, cyclin D1 is up-regulated in co-cultured GC cells with *H. pylori* infection[24].

*Tp53*, the guardian of human genome, is a tumor suppressor gene that is commonly mutated in all types of human cancer. *TP53* gene mutation is observed in GC[25]. Moreover, P21Waf1/Cip1, as a target for p53, binds to cyclin A-CDK2 and cyclin D1-CDK4 complexes and inhibits their function. Loss of P21Waf1/Cip1 expression has been reported in the 60% of GC tissues. Moreover, the underexpression of P21Waf1/Cip1 is correlated with tumor invasiveness and metastasis, as well as poor prognosis in GC[26]. Besides, down-regulation of p27Kip1, a CDK inhibitor, has been observed in GC, and its down-regulation is correlated with advanced stages and invasiveness of the tumor[27].

P16 is a regulator of cell cycle that causes G1 phase arrest by the inhibition of CDK4 and CDK6. The expression of P16 is observed in tissues and serum of GC patients, while its expression is not detected in normal tissues and sera. *P16* DNA methylation can be used as a serum biomarker for early detection of GC[28].

#### Notch signaling

Notch signaling is an important pathway in tumorigenesis through the regulation of cell proliferation, apoptosis, and differentiation. Jagged1 is a ligand of Notch signaling. After binding Jagged1 to the Notch receptor, Notch1 receptor intracellular domain is cleaved by matrix metalloproteinase (MMP) and Y-secretase and consequently translocates into the nucleus to activate transcription machinery[29].

*H. pylori* infection can induce Notch signaling. Moreover, jagged1 expression is associated with aggressiveness of GC. Notch signaling induces expression of the cyclooxygenase-2 (COX-2) through the binding of the Notch1 receptor intracellular domain to the Cox-2 promoter, which results in GC progression[30]. The expression of Notch1 is detected in human GC, especially in well-differentiated intestinal type[31]. Furthermore, up-regulation of Notch1, Notch3, Jagged1, and Jagged2 are significantly correlated with the intestinal type of GC[32]. In addition, inhibition of Notch signaling pathway in GC leads to the activation of *PTEN*, which consequently induces G2/M cell cycle arrest[33]. Overexpression of Notch signaling target genes, such as *HEY1* and *HEY2*, has been reported in ESCC with significant correlation to the different indices of poor prognosis, including stage of tumor progression and lymph node metastasis[34].

#### Hippo signaling

**Fig. 2:**
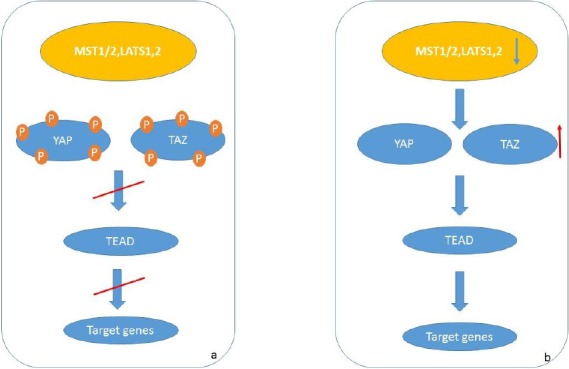
Hippo pathway. (a) During signaling pathway; the upstream components (MST1/1, LATS1/2) phosphorylate the downstream components and result in inactivation of pathway. (b) During GC; the expression of MST1/2 and LATS1/2 decreased and failed to phosphorylate YAP/TAZ. YAP/TAZ translocates to the nucleus and binds to the TEAD, resulting in transcription of target genes.

The Hippo signaling pathway is a key element in cell growth and organ size, as well as in the homeostasis of the gastrointestinal tissues. Moreover, dysregulation of Hippo pathway is associated with initiation, development, and distant metastasis of GC[35]. The main components of this pathway are MST1/2, LATS1/2, Mob1, YAP1, and TAZ1. MST1/2 phosphorylates and activates LATS1/2 and Mob1. Then LATS1/2 phosphorylates YAP1 and TAZ and increases 14-3-3 binding to phosphorylated YAP1/TAZ, leading to the oncogenic accumulation of the YAP1/TAZ in the cytoplasm. The unphosphorylated YAP1/TAZ translocates to the nucleus and binds to the TEAD1-4 transcription factors to induce transcriptional activity for cell growth and differentiation[36] (Fig. 2).

While the down-regulation of upstream components of Hippo pathway, such as MST1/2 and LATS1/2, is detected in GC, up-regulation of YAP1 that is the main downstream component is observed in high-grade dysplasia and metastatic GC[37]. Moreover, YAP1 is negatively regulated by tumor suppressor microRNAs, including miR-15a, miR-16-1, and miR-506 in GC[38].

The gain of function mutation in RhoA, an activator of YAP1, has been detected in diffuse type of GC[39]. *TEAD4* gene, as the main transcription factor of this pathway, is significantly hypo-methylated, and its overexpression is observed in GC[40]. Furthermore, the expression of TAZ, another key effector of the Hippo pathway, is associated with the overexpression of β-catenin and poor prognosis in GC[41].

### Epithelial-mesenchymal transition (EMT)

EMT is a cellular process that normally occurs during heart morphogenesis, mesoderm and neural crest formation, embryogenesis, wound healing, as well as fibrotic disease and cancer[42]. There are three types of EMT process. The type one of EMT is involved in generating mesenchymal cells; these cells can undergo a MET process to produce secondary epithelial cells. Actually, this type of EMT plays a role during embryogenesis and organ development. The type two of EMT involves in wound healing and tissue reconstruction and organ fibrosis. Moreover, type two is an essential factor during inflammation. The type three of EMT has a key role in neoplastic cells; these cells have enormous genetic and epigenetic changes, especially in oncogenes and tumor suppressor genes. Those neoplastic cells that undergo the type three of EMT may invade and metastasize, thereby leading to cancer progression[43]. Through the EMT process, cell phenotype changes from epithelial to mesenchymal. Indeed, epithelial cells lose their cell-cell adhesion, alter their polarity, rearrange their cytoskeleton and become isolated[44].

During EMT process, the down-regulation of E-cadherin, which is essential for the cell adhesion and is expressed at the surface of the epithelial cells, occurs. Moreover, the overexpression of N-cadherin, which is expressed in the mesanchymal cells, is another important event in EMT[45]. Other proteins such as FSP1, β-catenin, α-SMA, extracellular matrix (ECM), and cytoskeleton proteins are also determinant in EMT progress[46]. Besides, WNT5A induces EMT-related genes in GC and probably regulats EMT process[47]. In addition, paired-related homeobox 1 is up-regulated in GC. Additionally, PRPX1 induces EMT through the activation of the Wnt/B-catenin pathway[48]. Furthermore, the overexpression of the *Twist1*, a regulatory protein of EMT, and Vimentin as well as PDCD4 and E-cadherin downregulation have been detected in GC samples. Moreover, CagA transfection into GC cells can activate TWIST1 and Vimentin. Besides, CagA can decrease the expression levels of the E-cadherin through the down-regulation of the PDCD4[49]. Down-regulation of the Twist1 is associated with the up-regulation of the E-cadherin, suggesting that Twist1 induces EMT in GC[50]. Furthermore, the expression of the erythropoietin-producing hepatocellular A2 is positively associated with the EMT markers in GC[51]. Moreover, Fas signaling induces EMT and increases metastasis in GC. During the progression of GC, the overexpression of the FasL, phospho-GSK-3β, Snail, and B-catenin is observed[52].

The overexpression of the transforming growth factor beta (TGF-β1), Twist1, Snail, Slug, and Vimentin, as well as CD44, which is a CSC marker, is found in patients with dysplasia or early GC. Moreover, the expression levels of E-cadherin, an epithelial marker, decreased. Furthermore, eradication of the *H. pylori* infection decreased the levels of the TGF-β1, Twist, Snail, Slug, and Vimentin, while the levels of the E-cadherin increased. These data suggests that *H. pylori* may induce EMT through TGF-β1[53].

EMT is a key factor in gastric tumorigenesis. GC stem cells are significantly correlated with the expression of the EMT activating transcription factors. Moreover, CD44 expression is significantly associated with the expression of the Snail-1, ZEB-1, and E-cadherin in GC[54]. Overexpression of *MAML1* and *TWIST1* is significantly correlated with lymph node metastasis in ESCC patient[55]. Furthermore, the expression levels of *TWIST1* and *SNAIL* genes are significantly correlated with invasion in ESCC cell line KYSE-30 where ectopic expression of *TWIST1* results in the significant down-regulation of *SNAIL*[56]. Some of the important factors of EMT have been summarized in Table 4.

**Table 4:** EMT factors

Gene	Function	Cancer	Reference
*E-Cadherin*	Cell adhesion Expressed in epithelial cell	During EMT, the loss of E-cadherin expression occurs.	[122]
*N-Cadherin*	Expressed in mesenchymal cells	Gain of N-Cadherin expression during EMT occurs.	[122]
*TWIST1*	A transcription factor induces EMT and increases metastasis	Overexpression in GC and EMT happens.	[123]
*SNAIL*	Transcription factor that controls EMT during embryogenesis and tumorigenesis	Its expression is associated with tumorigenesis in GC during EMT.	[124]
*ZEB-1*	A transcription factor that induces EMT and metastasis	It overexpressed in GC.	[125]
*Vimentin*	Mesenchymal marker in EMT	Its overexpression is observed in GC during EMT.	[126]
*Slug*	Regulator of EMT	It overexpressed in GC.	[127]

### Matrix metalloproteinase

MMPs) break down the components of the ECM. MMPs and their tissue inhibitors act in tumor invasion and metastasis. The levels of the MMPs and tissue inhibitors increased in GC[57]. Besides, the overexpression of MMP9 in GC is associated with tumor invasion, and its serum level has a relation with the lymph node metastasis. Therefore, this data suggests that MMP9 is a novel biomarker for diagnosis and prognosis of GC[58]. The overexpression of *MMP2*, *MMP7*, and *MMP9* has also been observed in GC[59]. Interestingly, the expression of MMP1 is associated with the metastasis of GC cells[60]. Expression of the integrin αvβ6, which is an epithelial-specific receptor for fibronectin (an ECM protein), is associated with MMP9 in GC[61].

### TGF-β signaling pathway

TGF-β signaling pathway involves in many cellular processes such as cell growth, cell differentiation, and apoptosis. This pathway has many ligands, including TGF-β, activin, inhibin, bone morphogenetic proteins, Nodal, and others[62]. Furthermore, this pathway has two receptors: type I and type II, which are serine/threonine kinase receptors. During signaling, the ligands bind to the type II receptor where it catalyzes and phosphorylates the type I receptor. Then type I receptor phosphorylates SMADs proteins such as SMAD2/3; these proteins heterodimerize with SMAD4 and translocate into the nucleus to activate the transcription of target genes[63]. Dysregulation of the components of this pathway occurs in GC. The overexpression of TGF-β1 is detected in GC[64]. Besides, its expression is associated with lymph node metastasis[65]. Moreover, the polymorphism -509C>T in the promoter region of *TGF-β1* has a connection with worse prognosis in GC[64]. RUNX3 is one of the target proteins in TGF signaling that is a defining factor in induction of apoptosis in GC cells and its inactivation has been found in GC[66]. Furthermore, *H. pylori* infection leads to the methylation of *RUNX3* and inhibits its expression in GC[67]. Moreover, inactivation of *SMAD4* has been reported in GC[68]. Additionally, mutations in *TGFβRII* occur in GC tissues, which are likely the result of microsatellites’ instability. *TGFβRII* gene has 10 poly-A repeats that make them as hotspot regions for mutation[69]. Besides, mutations in *TGFβRI* are less frequent in GC, andits downregulation is associated with poor prognosis[70].

### Cyclooxygenase-2 and lipoxygenase (LOX) pathways

COX-2/ Prostaglandin E2 is one of the important pathways during gastric carcinogenesis. The COX enzymes, COX-1 and COX-2, are key effectors in prostaglandin synthesis. COX-1 has a function in the maintenance of the gastric mucosa integrity, while COX-2 is an inducible enzyme and can produce the prostaglandins. Prostaglandins are necessary for the reactions during the inflammatory processes. The normal mucosa of gastric produces COX-1, but the expression level of COX-2 is too low or undetectable. Moreover, COX-2 takes part in inflammation and carcinogenesis[71]. Many studies have reported the overexpression of COX-2 in GC[72-74]. Besides, the *H. pylori* infection may induce the expression of *COX-2* in GC. *H. Pylori* infection induces the *COX2* expression through p38 mitogen-activated protein kinase/activating transcription factor-2 signaling pathway in MKN45 GC cells[75]. Therefore, this pathway could be a novel therapeutic target for patients who have *H. pylori*-associated GC. Furthermore, *H. pylori* leads to the overexpression of vascular endothelial growth factor (VEGF) in MKN45 cells, which may be mediated by COX-2[76]. Moreover, the correlation between *COX-2* expression and *VEGF* expression has been reported in GC, suggesting the important role of prostaglandins in gastric carcinogenesis[77]. Additionally, COX-2 regulates the expression of Snail through Notch signaling pathway. The *COX-2* expression has an inverse correlation with the Notch1 expression in GC cells[78].

LOX pathway is an important pathway in producing leukotrienes and hydroxyeicosatetraenoic acids from arachidonic acid[79]. This pathway is also dysregulated during gastric carcinogenesis. In addition, 12-LOX is important during tumorigenesis. Its expression is found in GC cells, including AGS and MKN-28. Furthermore, 12-LOX regulates the apoptosis and cell proliferation in GC cells, and blocking the activity of 12-LOX leads to the inhibition of cell growth and activation of apoptosis[80,81]. Furthermore, the overexpression of LOX-5 has been reported in GC where its expression is associated with lymph node metastasis and TNM staging of the tumor[82]. Moreover, during *H. Pylori* infection, the activity of 5-LOX and the amount of 5-hydroxyeicosatetraenoic acid, which is the product of the function of 5-LOX on arachidonic acid, increased in GC cells[83]. Besides, the inhibition of 5-LOX led to the activation of apoptosis in GC cells[84].

### Epidermal growth factor receptor (EGFR), Human epidermal growth factor receptor 2 (HER2) signaling pathway

EGFR, a member of Erb-B family receptors, has a role in gastric mucosa proliferation and development of GC, and its overexpression is associated with poor prognosis in GC[85]. Furthermore, the overexpression and amplification of *HER2*, another member of ErbB family, has been detected in GC[86].

One of the downstream components of HER2 and EGFR pathways is Ras, an oncogenic GTPase that has three isoforms, including K-Ras, H-Ras, and N-Ras. Mutation in *K-RAS* gene has been detected in intestinal type of GC[87]. Moreover, mutations in *K-RAS* gene in *H. pylori*-associated chronic gastritis is more frequent in GC patients than those who did not have cancer. This finding suggests that *K-RAS* gene mutation is involved in the early stages of gastric carcinogenesis of the intestinal type[88]. Besides, fluorescent *in situ* hybridization study on gastric tumors, cell lines, and patients-derived xenografts shows the amplification of RTK/Ras components, including FGFR (fibroblast growth factor receptor) 2, HER2, and K-Ras[89].

### Nuclear factor-kB

NF-kB is a family of bipartite transcription factors that include NFKB1, NFKB2, c-Rel, RelA, and RelB. The common form of NF-kB in mammalian is RelA/NFkB1 dimer. Activation of this pathway occurs during inflammation. NF-kB normally binds to its inhibitor, inhibitory proteins of kB family (*IkB*), which leads to NF-kB being restricted in the cytoplasm. During inflammation, IkB kinase complex phosphorylates IkB, and then the degradation of IkB and activation of NF-kB occur[90]. *H. pylori* infection induces NF-kB activation in GC. Besides, *H. pylori* infection induces the expression of the pro-inflammatory cytokine IL-8 through the activation of the NF-kB[91]. HuR, a RNA-binding factor, is a direct transcript target of NF-kB and its activation in GC cell lines depends on phosphatidylinositol 3-kinase/ AKT signaling. HuR activation has proliferative and anti-apoptotic effects on GC[92]. Fructose-1,6-bisphosphatase-1 is an antagonist of the glycolysis process. The NF-kB is involved in glycolsis process through downregulation of FBP1 expression in GC[93]. Furthermore, the aberrant expression of NF-kB has anti-apoptotic effects and leads to drug resistant in GC[94,95].

### Treatment of gastric cancer

Surgery is the only curative treatment of GC, whereas perioperative and adjuvant chemotherapy, in addition to chemoradiation can improve the outcome of resectable GC with extended lymph node dissection.

According to the National Comprehensive Cancer Network (NCCN), the treatment of the early stages of GC guidelines includes endoscopic resection or complete surgical resection for long-term survival. Furthermore, in advanced stages of GC, the treatment includes preoperative chemotherapy, or chemoradio-therapy after surgery. The patients who have extended lymph node resection (D2) are recommended to have postoperative chemoradiation or chemotherapy. The recommendation for patients who have unresectable tumors is treating with fluoropyrimidine- or taxane-based chemoradiotherapy[96].

There are several genes with altered expression pattern in GC that can be a target for cancer-therapy (Table 5). Trastuzumab, a humanized anti-HER2 monoclonal antibody, is used against HER2-positive GCs[97]. Cetuximab, an anti-EGFR monoclonal antibody, cannot induce any response in GC when used alone. It is shown that VEGF and their receptors are overexpressed in GC[98]. In this regard, Ramucirumab, a fully human IgG1 antibody against VEGFR2, is now approved by FDA for the treatment GC[99].

**Table 5:** Genetic alteration targets for treatment of GC

Gene	Function	Expression in GC	Treatment	Reference
*HER2*	Regulation of cell growth and differentiation,	Over, Amp	Trastuzumab	[97]
*EGFR*	Cell growth, cell profilation, and cellular survival	Over, Amp	Cetuximab Nimotuzumab	[103,104]
*MET*	Embryogenesis, cellular survival, and cellular migration	Over, Amp	Onartuzumab	[102]
*HGF*	Regulation of cell motility and cell growth, morphogenesis of numerous cells and tissues, and angiogenesis	Over	Rilotumumab	[101]
*VEGF*	Angiogenesis, bone formation, hematopoiesis, wound healing, and development	Over	Bevacizumab	[128]
*VEGFR2*	Tyrosine kinase receptor, angiogenesis, embryonic hemopoiesis, regulation of cell profilation, and organization of ECM	Over	Ramucirumab	[99]
*FGFR2*	Cell division, cell growth, formation of blood vessels, wound healing, and embryonic development	Over, Amp	AZD4547	[100]
*IGFR*-IR	Cell growth	Over	Figitumumab	[129]
*NF-κB*	Immune response to infection	Over	Bortezomib	[130]
*mTOR*	Cell growth, cell proliferation, and cell cycle	Over	Everolimus	[131]
*MMPs*	Degradation and destruction of ECM	Over	Marimastat	[132]

Over, overexpression; Amp, amplification

AZD4547, as a selective ATP-competitive receptor tyrosine kinase inhibitor of FGFR, is effective against patients who have amplification of FGFR2[100]. Furthermore, hepatocyte growth factor (rilotumumab, a fully human IgG2 monoclonal antibody against HGF), hepatocyte growth factor receptor (onartuzumab, humanized monoclonal antibody directed against HGFR), and EGFR (cetuximab, an anti-EGFR monoclonal antibody and a nimotuzumab that is a humanized monoclonal IgG_1_ antibody to EGFR), are also the targets of treatment in GC[101-104].

Here, we summarized multiple pathways involving in GC carcinogenesis. A better understanding of molecular mechanisms of GC progression and development, as well as crosstalk between signaling pathways can help to identify new targets for anticancer drugs. Although many studies have been done on GC, the mechanism of GC carcinogenesis is still unclear. Understanding the molecular processes of GC could help to design more efficient genetic studies. With the novel technology advances, it will be easier to find new and useful targets in signaling pathways; these targets will be a potential marker for the early diagnosis and treatment of GC. Therefore, the management and the efficiency of treatment in patients with GC will be improved in future.
